# Manual of Physiology with Practical Exercises

**Published:** 1910-12

**Authors:** 


**Manual of Physiology with Practical Exercises.** By **G. N. Stewart** M.D., Professor of Experimental Medicine in Western Reserve University Cleveland. Sixth edition. Small octavo, pp. 1084. Illustrated. New York: William Wood & Co. 1910. (Cloth, $5.00.)

The plan upon which this laboratory work is built is that long followed by the author in teaching physiology to his classes in this country and in Europe. The systematic portions of the book are so arranged that it can be used equally well independently of the practical work. The volume aims at being in itself a complete exposition of the subject, adapted to the requirements of the student of medicine. In the present edition the book has been extensively revised, and in many parts rewritten. A considerable amount of new matter has been added, increasing to quite an extent the size of the volume. J. A. R.

